# Maternal cortisol stimulates neurogenesis and affects larval behaviour in zebrafish

**DOI:** 10.1038/srep40905

**Published:** 2017-01-18

**Authors:** Carol Best, Deborah M. Kurrasch, Mathilakath M. Vijayan

**Affiliations:** 1 Department of Biological Sciences and University of Calgary 2500 University Drive NW Calgary, Alberta, T2N 1N4, Canada; 2 Department of Medical Genetics, University of Calgary 2500 University Drive NW Calgary, Alberta, T2N 1N4, Canada

## Abstract

Excess glucocorticoid transferred from stressed mother to the embryo affects developing vertebrate offspring, but the underlying programming events are unclear. In this study, we tested the hypothesis that increased zygotic glucocorticoid deposition, mimicking a maternal stress scenario, modifies early brain development and larval behaviour in zebrafish (*Danio rerio*). Cortisol was microinjected into the yolk at one cell-stage, to mimic maternal transfer, and the larvae [96 hours post-fertilization (hpf)] displayed increased activity in light and a reduction in thigmotaxis, a behavioural model for anxiety, suggesting an increased propensity for boldness. This cortisol-mediated behavioural phenotype corresponded with an increase in primary neurogenesis, as measured by incorporation of EdU at 24 hpf, in a region-specific manner in the preoptic region and the pallium, the teleostean homolog of the hippocampus. Also, cortisol increased the expression of the proneural gene *neurod4*, a marker of neurogenesis, in a region- and development-specific manner in the embryos. Altogether, excess zygotic cortisol, mimicking maternal stress, affects early brain development and behavioural phenotype in larval zebrafish. We propose a key role for cortisol in altering brain development leading to enhanced boldness, which may be beneficial in preparing the offspring to a stressful environment and enhancing fitness.

Glucocorticoid signaling is important for vertebrate development, including brain development and behavioural programming^1^. Abnormal levels of glucocorticoids during early development can alter developmental programming, often with detrimental effects in mammals^1^. In fish, maternal cortisol transferred to the oocytes is the only source of steroid until the hatching stage and is essential for early development, including maturation of the cortisol stress axis^2^,^3^,^4^. Stressed mothers transfer excess cortisol to the oocytes^5^, and this affects developmental programming, including hypothalamus-pituitary-interrenal (HPI) axis functioning in zebrafish (*Danio rerio*)^4^, suggesting improper brain development^3^.

In zebrafish, neurogenesis as measured by the earliest differentiation of neurons from progenitors in the brain occurs during the segmentation period at approximately 18 hpf^6^,^7^,^8^,^9^. Various basic helix-loop-helix (bHLH) transcription factors, including those in the neuronal differentiation (*neurod*) family promote differentiation of neurons in specific regions in the developing vertebrate brain^10^,^11^,^12^. *Neurod4* is involved in primary neurogenesis in the midbrain, hindbrain and retina of zebrafish^13^,^14^. In addition, *orthopedia*, a homeodomain transcription factor, is another key marker of neurogenesis and is specific to development of dopaminergic and neuroendocrine cells, including corticotropin releasing factor (CRF)neurons^15^,^16^. Transcript abundance of these neuronal markers was disrupted by lack of glucocorticoid receptor (GR) signalling during zebrafish early development^17^, leading to the proposal that cortisol may play a role in normal brain development^4^.

Glucocorticoid-mediated changes in brain development may also be reflected in altered behavioural responses^1^. The zebrafish larvae is well suited to behavioural analyses due to high offspring yield and the ability to simultaneously assess the behaviour of many replicate individuals using high-throughput behaviour-screening systems^18^. As a result many early larval behavioural responses, including locomotor activity, have been well characterized^19^. The locomotor response to alternating periods of light (lower activity) and dark (higher activity) provides information on not only locomotor activity, but also visual ability to detect light changes, and freezing behaviour (fright or anxiety) in response to sudden light onset^20^,^21^. This response to light/dark changes is highly conserved and has been observed in larval zebrafish^20^,^21^,^22^, fifth-instar larvae of migratory locusts^23^, and in rodents^24^,^25^. Thigmotaxis is another behavioural assay used to assess anxiety in rodents^26^ and was also adapted for use with zebrafish^27^. Thigmotaxis measures the propensity for an animal to remain close to the walls of the test area, as opposed to actively exploring the environment. Few studies to date have explored the links between physiological stress/cortisol stimulation during early development and behaviour later in life in zebrafish^18^,^28^,^29^.

Since the brain coordinates both the cortisol stress axis as well as behavioural responses, altered neurogenesis in response to maternal stress (e.g., elevated cortisol deposition) may be a mechanism for developmental programming that results in long-term changes in behavior, but this has not been tested yet. We hypothesized that increased zygotic deposition of cortisol would disrupt neurogenesis and compromise larval behaviour in zebrafish. To address this, 1-cell stage embryos were microinjected with cortisol to mimic elevated maternal deposition, and the resulting effect on neurogenesis and larval behaviour were assessed.

## Results

### Cortisol Developmental Profile

**Figure 1:**
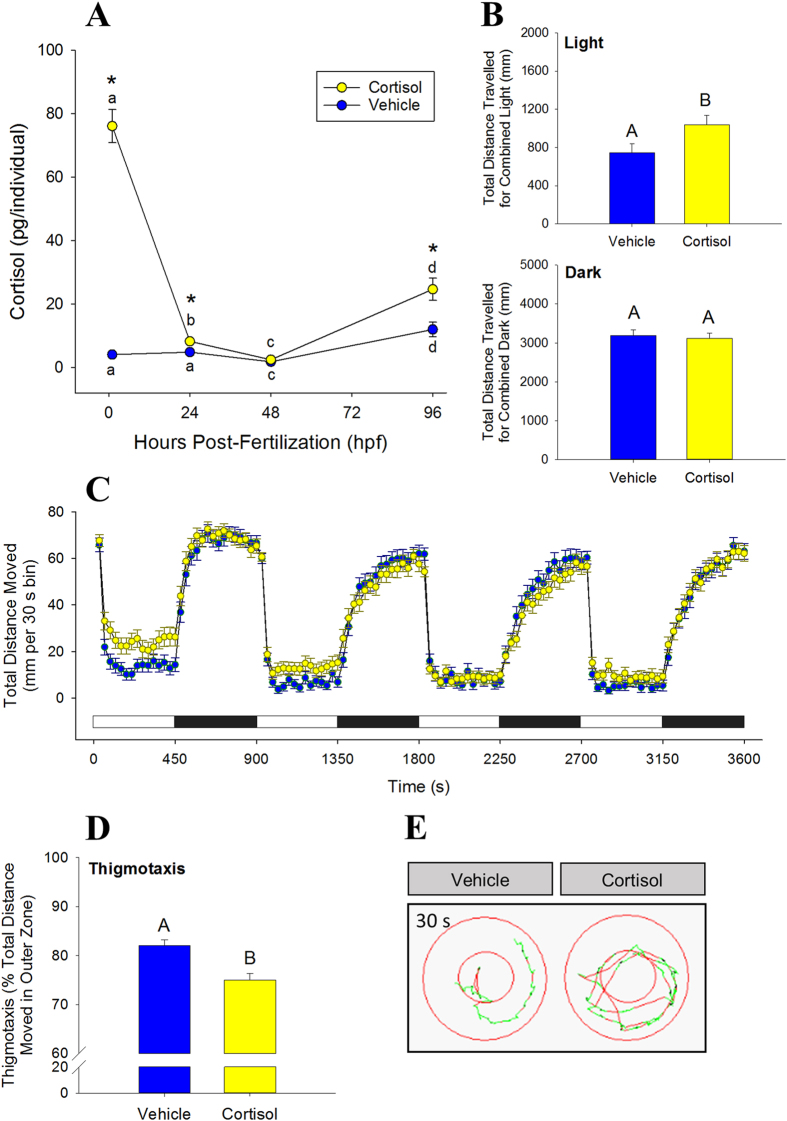
Excess zygotic cortisol content affects larval behaviour. (**A)** Cortisol profile in injected zebrafish. Embryos were microinjected with either vehicle (control) or 75 pg cortisol [Means ± SEM, n = 4–9 (pools of 20)]. A significant interaction was detected (two-way ANOVA/Tukey, P < 0.001). Significant time effects within treatment groups are indicated by different lowercase letters; significant treatment effects within time points are indicated by asterisks. (**B)** Total activity in light and dark period. Activity of 96 hpf larvae expressed as total distance travelled in 30 m. Cortisol levels corresponds to those at 96 hpf (see A). Means ± SEM (n = 72). Different letters indicate significant differences (Student’s t-test, unpaired). (**C)** Mean activity in light and dark. Activity (96 hpf) is expressed as total distance moved during each 30 s recording bin. The total recording period was 1 h with alternating light periods of 7.5 min each, indicated by the light and dark bars above the x-axis. Means ± SEM (n = 72). (**D)** Thigmotaxis. Arenas (wells) were divided into inner and outer zones, and the propensity to stay close to arena wall (96 hpf) is expressed as % of total distance travelled in 30 min that occurred in the outer zone. Means ± SEM (n = 48). Different letters indicate significant differences (Student’s t-test, unpaired). (**E)** Representative path for thigmotaxis. Inner and outer zones shown, in addition to a representative 30 s travel path for one larvae in each treatment group.

Cortisol levels in the vehicle-injected embryos decreased over the first 48 hours post-fertilization (hpf), and then increased following hatching. A mean initial cortisol level of 4.1 ± 0.7 pg per embryo was present in the control embryos at 1 hpf, and this dropped significantly (P = 0.028) to 1.8 ± 0.5 pg/embryo by 48 hpf (Fig. 1A). By 96 hpf, cortisol levels in the larvae increased significantly (P < 0.001) to a mean of 12.0 ± 2.3 pg/larvae. Embryos injected with cortisol showed significantly higher initial cortisol levels at 1 hpf (76.1 ± 5.7 pg/embryo) compared to the controls. At 24 hpf, values in the cortisol-injected group remained significantly higher than the controls (P = 0.037). By 48 hpf, the lowest point of the developmental cortisol profile, cortisol-injected values were no longer significantly different compared to the control (P = 0.204). At 96 hpf, cortisol levels in the cortisol-injected group had increased once again, and were significantly higher than controls (P = 0.003).

### Behaviour

At 96 hpf all larvae displayed elevated locomotor activity in the dark and decreased activity during periods of light (Fig. 1C). Activity was measured as total distance travelled in mm per 30 s recording bin, and was approximately 6-fold lower in bright light than in the dark (Fig. 1C). When all light periods of the same type are combined for the 1 h protocol (Fig. 1B) the cortisol group has significantly higher activity, in terms of distance travelled. There was no difference in total distance traveled between the two groups when measured in the dark. Thigmotaxis was significantly lower in the cortisol group than the controls, indicating that larvae with increased zygotic cortisol deposition spent less time around the perimeter of the well than the vehicle-injected fish (Fig. 1D).

### Neurogenesis

**Figure 2:**
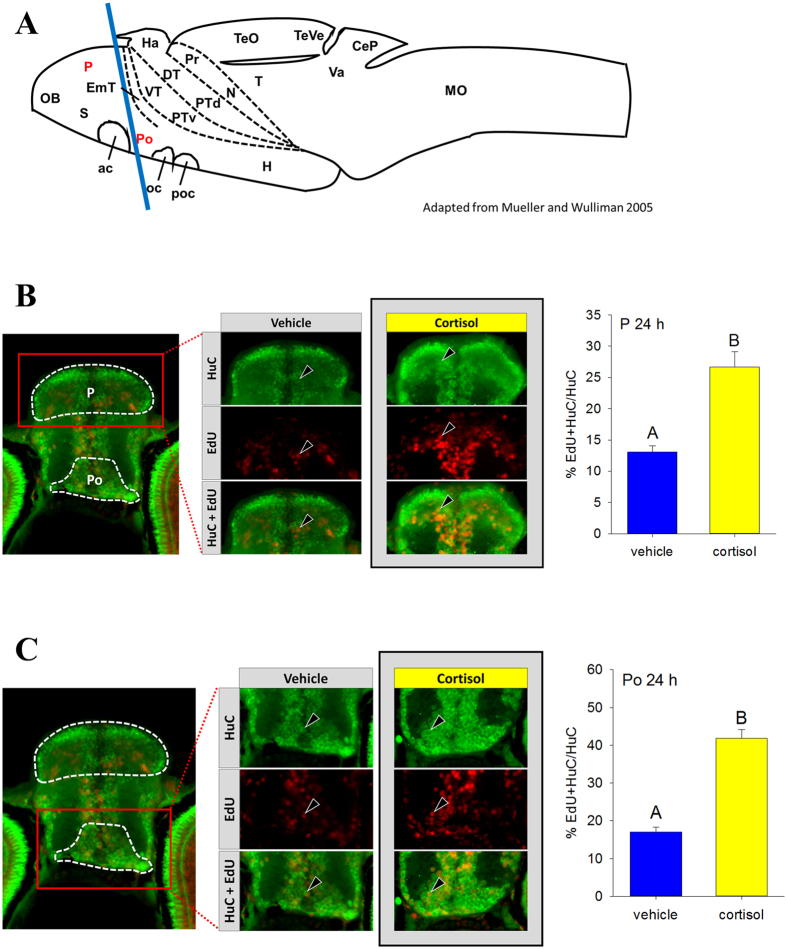
Excess zygotic cortisol content affects neurogenesis. (**A**) A schematic of zebrafish brain showing the regions used for assessing neurogenesis. The regions, including P (pallium) and Po (preoptic region), highlighted in red were, used for assessing neurogenesis. Position of transverse section selected for further analysis is shown by a blue line. See SI Table 1 for full list of abbreviations. Adapted from Mueller and Wulliman^66^. Neurogenesis in zebrafish pallium (**B**) and preoptic region (**C**). Larvae were pulsed with EdU at 24 hpf and raised until 120 hpf. A representative image contains outlines of the regions of interest (white dashed line). Representative sections for vehicle and control groups are shown, with staining for HuC (green), EdU (red) and both merged. Examples of cells co-labelled with EdU and HuC are indicated with arrowheads. Cells were counted by region and total number of new neurons (EdU + HuC) at 24 hpf and was normalized to total number of neurons (HuC). Means ± SEM (n = 3–5 larvae). Different letters indicate significant differences (Student’s t-test).

Neurogenesis at 24 hpf was measured by EdU pulse-labelling followed by sampling, immunostaining and imaging of the 120 hpf brain, specifically the transverse section indicated by the blue line in Fig. 2A (See SI Table 1 for brain regions). Neurogenesis was higher in response to excess cortisol exposure in both the pallium (P, Fig. 2B) and the preoptic region (Po, Fig. 2C), but not in the rostral hypothalamus (Hr, SI Fig. 1A and B), the posterior tuberculum (PT, SI Fig. 1A and C), and the dorsal thalamus (DT, SI Fig. 1A and D).

### Transcript Abundance

**Figure 3:**
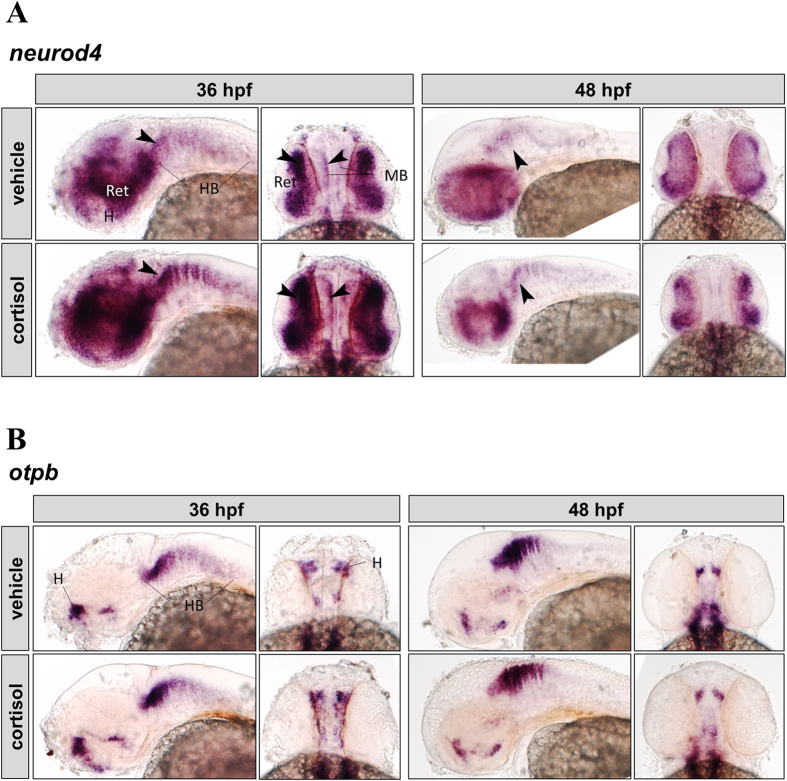
Cortisol effect on *neurod4* and *otpb* expression in zebrafish embryos. Whole-mount *in situ* hybridization of *neurod4* (**A**) and *otpb* (**B**). Representative images (left, lateral view; right, dorsal view) are shown for each treatment (vehicle: top, cortisol: bottom) and time point (36 hpf: left, 48 hpf: right) on each panel. Regions indicated on vehicle controls (H, hypothalamus; Ret, retina; MB, midbrain; HB, hindbrain). Arrowheads indicate regions with differential expression; see text for details.

Spatial distribution of key proneural transcript abundance in the intact brain (36 and 48 hpf) was assessed by whole-mount ISH (Fig. 3). Transcripts of neuronal differentiation 4 (*neurod4*) were found in the eye (retina), midbrain, and in a segmented pattern in the hindbrain at both 36 and 48 hpf, but with visibly reduced expression at 48 hpf compared to 36 hpf in both groups (Fig. 3A). The spatial transcript abundance of *neurod4* at 36 hpf was stronger in the cortisol group, particularly in the retina and to a lesser extent along the midbrain and hindbrain regions (Fig. 3A, 36 hpf, arrowheads). At 48 hpf the cortisol group has higher expression relative to controls at the midbrain-hindbrain boundary (Fig. 3A, 48 hpf, arrowheads).

Transcripts of orthopedia b (*otpb*) appear in the hindbrain, and dorsal hypothalamus at both 36 and 48 hpf (Fig. 3B). Spatial transcript abundance of *otpb* appears unchanged by cortisol treatment (Fig. 3B).

## Discussion

Our results demonstrate that elevated zygotic cortisol levels, mimicking cortisol transfer from stressed mothers to offspring, enhanced boldness in larval zebrafish as measured in behavioural assays. This behavioural phenotype also corresponded with increased neurogenesis at 24 hpf, implicating for the first time a role for excess cortisol deposition in response to maternal stress as a mediator of brain development and function in zebrafish. The cortisol profile observed in the zebrafish supports earlier studies showing a decrease in maternal deposits during embryogenesis and an increase in cortisol levels post-hatch in this species^2^,^3^,^30^. The importance of maternal cortisol deposition, its utilization during embryogenesis until hatch and its subsequent biosynthesis, is conserved among fish species^31^,^32^,^33^,^34^. There is a growing body of evidence that this maternal cortisol deposition, acting through GR, is essential for zebrafish development^3^,^17^,^35^,^36^,^37^,^38^.

Maternal stress and the attendant rise in cortisol levels can increase the deposition of this steroid to the oocytes^5^. To mimic this deposition, embryos were injected with 75 pg cortisol, a level similar to that seen in embryos following a five-day fasting stressor in mothers (64.7 ± 14.1 pg/egg)^39^. When mothers are fed exogenous cortisol, embryos showed elevated cortisol content (35.3 ± 9.9 pg/egg)^5^. However, zebrafish are asynchronous breeders and show variability and time-dependent changes in cortisol incorporation into the oocyte, and the values represent the day where zygotic cortisol levels were highest over a ten-day period^5^,^39^.To our knowledge, these are the only studies that have measured mother-to-zygote cortisol transfer in zebrafish. Microinjection of cortisol sustained significantly higher levels of this hormone only until 24 hpf, and this supports the decrease in cortisol after microinjection seen previously in zebrafish^38^. The return of all elevated cortisol deposits back to control levels by 48 hpf (hatch) illustrates the importance of maintaining low cortisol levels during this early developmental window. While the mechanism(s) is unclear, a recent study suggested a role for ATP-binding cassette transporters in the rapid cortisol clearance in threespine stickleback (*Gasterosteus aculeatus*) embryos^40^. Another possibility is the enzyme 11βHSD2, which converts cortisol to inactive cortisone and contains putative glucocorticoid response elements (GRE) in its promoter region^41^. This enzyme plays a role in countering exposure to cortisol excess in zebrafish ovarian follicles^5^, but its role in embryos remains to be elucidated.

Here, elevated zygotic cortisol content clearly affected the larval behaviour later in life, suggesting long-lasting effects of early exposure to elevated cortisol levels. We employed two behavioural tests, locomotor activity in light and dark and thigmotaxis, on 96 hpf larvae to assess the effect due to excess cortisol exposure of embryos. Measurement of locomotor activity is often performed as an initial assessment of neuromuscular function, anxiety, and/or exploratory behaviour^42^. Typically zebrafish display hyperactivity in the dark and hypoactivity (freezing) in the light^21^ and this was supported by our results. The freeze response, a behaviour that is generally interpreted as an aversive response to sudden light onset, dissipates with time^21^ as seen in the present study. Hyperactivity in the dark was unaffected by elevated zygotic cortisol; however, there was an attenuation of the freeze response to light in the larvae hatched from cortisol-enriched embryos. An animal’s behavioural reaction to risks or novel stimuli determines its boldness^43^, and bold animals are better at escaping from a predation risk^44^. Therefore, the higher activity level of larvae raised from cortisol-enriched embryos in response to sudden light suggests a phenotype of increased boldness. However, this could also be interpreted as a panic response to stress, but this appears less likely given that thigmotaxis was reduced in larvae raised from cortisol-enriched embryos. Bold individuals readily explore open areas despite risks, while shy or anxious individuals are more thigmotactic^27^,^43^. Although similar studies in fish are limited, zebrafish bathed in dexamethasone, a synthetic glucocorticoid, for the first 5 d of development displayed increased boldness at 84 dpf^45^, implying that the phenotype of increased boldness may persist into adulthood. Also, larvae raised from coho salmon (*Oncorhynchus kisutch*) eggs exposed to exogenous cortisol displayed increased boldness at 3 months in response to a simulated predation event^44^. However, a similar egg exposure regimen to cortisol in brook trout (*Salvelinus fontinalis*) did not lead to altered boldness in a novel environment after 6 months of rearing^46^, suggesting that the behavioural programming effects may be context- and species-dependent.

While links between developmental glucocorticoid exposure and behaviour in fish are sparse, such links exist in other vertebrates^1^. For instance, prenatally stressed male guinea pigs displayed increased thigmotaxis as juveniles and adults^47^. Reduced exploratory behaviour of offspring has been observed in rats subjected to maternal dexamethasone (Welberg *et al*.^48^) or to prenatal restraint stress (Laloux *et al*.^49^, Zohar *et al*.^50^). Birds injected with corticosterone into the yolk displayed either no effect (Rubolini *et al*.^51^) or increased fearfulness in chicks, though this could be modified by handling exposure (Janczak *et al*.^52^). Based on these studies, the potential for developmental programming by stress hormone is evident, although factors such as timing and magnitude of glucocorticoid elevation renders interspecies comparisons difficult. In the present study, zebrafish larvae that experienced elevations in zygotic cortisol displayed behavioural changes indicative of increased boldness, and this may be a compensatory programming of behaviour mediated by excess cortisol passed from the mother to the offspring during early development. However, as the mechanisms behind these behavioural changes are not known, we hypothesized that this may be associated with altered brain development by glucocorticoids as reported in mammalian models^53^,^54^,^55^,^56^,^57^,^58^. Primary neurogenesis is a highly regulated process^11^, and its disruption during development by excess glucocorticoid exposure may interfere with nervous system function, in turn affecting downstream biological functions.

Teleost brain structures are fairly conserved with respect to other vertebrates^59^ and signalling pathways during early neurogenesis also appear to be conserved^11^. A noteworthy difference is that zebrafish display a much more widespread capability throughout the adult brain for neurogenesis^60^. In the present study neurogenesis did not change in response to excess zygotic cortisol content in the dorsal thalamus, posterior tuberculum and the rostral hypothalamus at 24 hpf, when rates of primary neurogenesis are highest^61^. However, neurogenesis in the pallium and the preoptic area at 24 hpf was significantly higher. In fish, the pallium contains homologues of the mammalian hippocampus, isocortex, basolateral amygdala and and piriform cortex^62^ and is involved in the control of behaviours, including emotional and spatial learning^63^. The preoptic area contains CRF neurons projecting to the pituitary and is the principal site for integration of signals controlling HPI axis activation^64^. Therefore, changes in neurogenesis in these regions are particularly relevant in the context of the behavioural changes seen in the current study, as well as the stress response changes seen in a previous study^4^. Altered early neurogenesis has been linked to behavioural phenotypes, including changes in activity level, as seen with early exposure to bisphenol A (BPA) in mice at 3–15 weeks old^65^ and zebrafish at 120 hpf^61^. Given these findings, our results suggests that altered neurogenesis in these specific brain regions may be involved in the behavioural phenotype (present study) and the impairment of larval stress performance seen with excess zygotic cortisol deposition^4^, underscoring an altered developmental programming of the progeny due to maternal stress in zebrafish.

This increase in neurogenesis is in contrast to what has been generally observed in rats exposed to prenatal stress^55^,^56^. However, one study comparing mild and severe prenatal stress in rats showed increased neonatal hippocampal neurogenesis in the offspring of mothers subjected to mild restraint stress^66^. Cortisol signalling involves GR and the mineralocorticoid receptor (MR) activation in zebrafish^3^, but most studies have examined the role of GR during early development. For instance, transcriptomic analysis following *gr* knockdown by morpholino during development predicted increased neuronal proliferation at 36 hpf^17^, implicating GR in the control of early neurogenesis in the zebrafish. *Neurod4* and *otpb* transcripts measured in the same study were significantly increased at 24 and 36 hpf^17^, leading us to propose a reduction in these transcripts in response to excess cortisol stimulation. However, in the present study, transcript levels of *neurod4* appeared to be higher throughout the larval brain of cortisol treated zebrafish, suggesting that MR signalling may also be playing a role in neurogenesis. While it is unclear whether *neurod4* is acting directly or in conjunction with other developmental pathways to increase neurogenesis, this is a potential mechanism by which cortisol may have a stimulatory effect on primary neurogenesis in zebrafish and warrants further study.

In conclusion, our results demonstrate that excess cortisol levels in the embryo, mimicking elevated maternal deposition during stress, affect larval behaviour in zebrafish. The behavioural phenotype, enhanced boldness, seen with cortisol treatment corresponds with increased neurogenesis in select brain regions, including the hippocampal analogue in fish and the preoptic region. We hypothesize that increased neurogenesis may underlie the behavioural phenotype seen in the larvae of cortisol-treated embryos. The mechanism leading to altered neurogenesis is not clear, but we hypothesize a role for cortisol in modulating the expression of proneural transcription factors, including *neurod4*. Overall, maternal stress and excess transfer of cortisol to the embryo affects developmental programming in zebrafish. In a broader evolutionary context, increased transfer of glucocorticoid from mother to offspring may act as a signal for altering programming events that may be beneficial to the offspring in order to cope with a stressful environment. For instance, in an environment where resources are limiting, increased boldness may increase fitness of the animal due to better foraging ability. On the other hand, in an environment with high predation, increased boldness may decrease fitness by increasing the risk of predation^43^,^67^. Therefore, the adaptive value of the observed phenotype may be context-dependent, but underscores cortisol as a maternal signal for programming offspring development leading to enhanced fitness and survival in a potentially stressful environment.

## Methods

### Animals

Zebrafish (Tupfel long fin strain) were held in a recirculating system in 10 L tanks (Pentair Aquatic Habitats, Apopka FL) on a 14:10 light:dark cycle. Water was maintained at 28 °C, 750 μS conductivity, and pH 7.6. Fish were fed twice daily consisting of Zeigler adult zebrafish food in the morning (Pentair Aquatic Habitats) and live *Artemia* (San Francisco Bay Brand, Inc.) in the afternoon. For breeding, fish were set up the previous evening in breeding traps, and eggs were collected the next morning. Embryos were maintained in E3 embryo medium (5 mM NaCl, 0.17 mM KCl, 0.33 mM CaCl_2_, 0.33 mM MgSO_4_, 0.1 ppm methylene blue as antifungal^68^,^69^) in 10 cm Petri dishes (60–100 embryos per dish), on a 14 h light :10 h dark cycle, with daily renewal of embryo medium (approx. 50%) and removal of any dead embryos or larvae. No significant treatment effect on mortality was observed. This maintenance continued until embryos or larvae were required for experiments, to a maximum of 5 dpf. All experimental protocols were approved by the Animal Care and Use Committee at the University of Calgary, and were in accordance with guidelines established by the Canadian Council for Animal Care.

### Microinjection of Cortisol

Injections were performed using a microinjection system and nitrogen gas (Narshige, Japan). Glass capillaries (Sutter Instruments, Novato, CA) were pulled into needles and trimmed prior to use. Cortisol (Sigma-Aldrich, St. Louis, MO, USA) solutions were prepared from ethanol stocks in sterile (0.2 μm filtered) water with 0.05% phenol red (Sigma-Aldrich) for visualization. On the day of injection, embryos were collected and maintained in system water until they were transferred to pre-warmed agarose injection trays. Following calibration (1 nL), pre-warmed cortisol solutions were delivered by microinjection into the yolk to mimic maternal deposition (vehicle control and 75 pg/egg). Injected fish were transferred into Petri dishes with E3 medium and raised as above. Samples of pooled embryos or larvae were collected at 1, 24, 48, and 96 hpf for measurement of cortisol, 24 hpf to assess neurogenesis, 36 and 48 hpf for *in situ* hybridization, and 36 hpf for quantification of transcript abundance.

### Cortisol Measurement

For cortisol quantification, pools of embryos or larvae (n = 12 or 20) were collected and all water was removed prior to snap freezing on dry ice. These pools of embryos or larvae were then partially thawed on ice and homogenized in 120 or 200 μl, respectively, of 50 mM Tris buffer, pH 7.5 with added protease inhibitors (Roche Diagnostics, Laval, QC, CAN). Samples were sonicated on ice to homogeneity and stored at −80 °C until use. Cortisol levels were measured using a competitive enzyme-linked immunosorbent assay as described previously for zebrafish^5^.

### Behavioural Analysis

Embryos were injected with vehicle or cortisol as above and raised to 72 hpf in the same standard conditions described above. All treatments and analyses were performed during the light portion of the 14 h light :10 h dark cycle, and at the same time of day. The larvae were transferred to clear multiwell plates (24 or 96) with lids (1 individual per well) and were allowed to acclimate overnight. Analyses were performed in an isolated room maintained at 28.5 °C. The movement of 4 dpf larvae was video captured and quantified using the ZebraBox infrared camera setup and the tracking extension of the ZebraLab software system (Viewpoint Life Sciences, Montreal, QC, CAN). In all behavioural protocols, the animal colour was set to black and the background-subtracted detection threshold was set to 20. This value represents a greyscale pixel intensity value, and any pixels darker than this threshold in the video are detected as the animal. The integration period (bin time) for movement data was set to 30 s. Data were processed and analyzed using Excel, and FastDataMonitor (Viewpoint Life Sciences).

### Activity in Light and Dark

For the light-dark response assay, 96 hpf larvae were subjected to short alternating periods of light and darkness, and their movement in response to these stimuli were recorded. The protocol parameters consisted of alternating periods of light and dark every 7.5 min (450 s). This 15 min cycle of light and dark was repeated four times for a total of 60 min. This was repeated over 3 trials (24 per treatment group per plate) for a total of 72 larvae per treatment. The total distance moved (in mm) was calculated per animal every 30 s integration period.

### Thigmotaxis

This behavioural assay is analogous to the open field test in rodents, used to assess anxiety by the tendency to remain close to the walls of the arena^70^. To test this, 72 hpf injected larvae were transferred in 0.5 ml of E3 embryo medium into each well of a 24 well plate and allowed to acclimate as above. Larger wells allowed space for creating two distinct inner and outer zones with the tracking software, each with widths ≥ one larval body length. The parameters for this assay consisted of these zone designations, no light, and a total duration of 30 min. This assay is run in the dark to induce strong activity levels, thus encouraging the animal to fully explore the environment^27^. This was repeated over 6 trials (8 per treatment group per plate) for a total of 48 larvae per treatment. The total distance moved (mm) over the entire duration of the 30 min assay was summed for each larva, for both the inner and outer zones. Thigmotaxis was calculated as the total distance moved by the animal in the outer zone, as a percentage of the total distance moved by the animal in total. This method corrects for individual differences in activity as recommended by Schnörr *et al*.^27^.

### Neurogenesis

Embryos were injected with cortisol or vehicle as above. To assess neurogenesis, embryos were pulse-labelled with the thymidine analogue ethynyl deoxyuridine (EdU) at 24 hpf, raised until 120 hpf and the brain sectioned and immunostained to identify neurons born at 24 hpf (n = 5) exactly as described previously^61^.

Briefly, injected fish were pulsed-labelled with EdU at 24 h by collecting 20 embryos in a 1.5 ml tube, aspirating off excess embryo medium and replacing it with prewarmed EdU solution (10 mM, Molecular Probes; C10338). Fish were gently washed and returned to their Petri dishes. At 5 dpf, fish were sacrificed and fixed in 4% PFA, washed, cryoprotected in 30% sucrose, embedded in OCT (optimum cutting temperature) compound (VWR Scientific), and stored at −80 until sectioning. Transverse 10 μm sections were taken through the brain using the Leica CM 3050 S cryostat (Leica Microsystems, Wetzlar, Germany). Brain regions were confirmed using the zebrafish brain atlas^71^ (see Fig. 2A; SI Table 1). Following antigen retrieval and permeabilization, sections were blocked with 5% normal goat serum for 1 h at RT, then incubated overnight at 4 °C in primary α-HuC (1:400, Molecular Probes; A21271). Secondary antibody was conjugated to Alexa Fluor 488 (1:400, Molecular Probes; A11001) and incubation was for 2 h at RT in dark. Slides were stained with DAPI (1:1000, Molecular Probes; D1308), and then the Click-iT reaction to detect EdU labelling was performed as per kit instructions (Molecular Probes; C10338). Slides were mounted and imaged by fluorescence microscopy.

Images were analysed using Fiji^72^. Images were overlaid to produce a multichannel image, then the Cell Counter plugin was used to count labelled cells in the specified regions of selected sections (DAPI, blue; HuC, green; and EdU, red – see Fig. 2B for representative sections). Counts of neurons born at 24 h (DAPI, HuC and EdU-positive cells) were normalized to total number of neurons (HuC-positive cells) in that region as described in ref. 61.

### Whole-Mount *In-Situ* Hybridization

Larvae (36 and 48 hpf; n = 3–4) were collected, fixed in 4% PFA and dehydrated in methanol for assessment of spatial transcript abundance by *in situ* hybridization. The transcripts detected were *otpb* and *neurod4*, and they play a role in primary neurogenesis.

Riboprobe templates were obtained by PCR using 48 h larval cDNA as template, and gene-specific primers with added T7 sites (SI Table 2). After PCR purification, *in vitro* transcription was performed by combining 200 ng purified PCR product, 2 μl 10x DIG labelling dNTP mix (digoxygenin, Roche Diagnostics), 4 μl 5× transcription buffer, 40 U RNase inhibitor (RiboLock, Thermo Scientific, Waltham, MA, USA), 40 U T7 RNA polymerase (Thermo Scientific) and nuclease free water to 20 μl final volume. The reaction was incubated for 2 h at 37 °C, volume was brought up to 50 μl with nuclease-free water and the riboprobe was purified using SigmaSpin columns (Sigma-Aldrich).

Whole mount *in situ* hybridization was performed as per the protocol of Kurrasch *et al*.^73^. Briefly, fish were rehydrated in PBS-T, followed by 30 min bleaching (0.5X SSC, 1% H_2_O_2_, 5% formamide), and permeabilization by proteinase K (36 hpf: 10 μg/ml × 30 min; 48 hpf 50 μg/ml × 8 min). Larvae were prehybridized for 2 h at 70 °C, followed by overnight hybridization with probe (1:100). Following a series of washes at 70 °C, larvae were incubated in blocking buffer (5% sheep serum) for 1 h at RT. The anti-DIG antibody (Roche Diagnostics) was diluted 1:1000 in blocking buffer and incubated overnight at 4 °C. Larvae were thoroughly washed and then stained with NBT/BCIP solution in staining buffer for 40 minutes in the dark. The reaction was stopped by washing and refixation in 4% PFA. Fish were transferred to 87% glycerol for clearing and imaged using a Nikon AZ-100 microscope.

### Statistics

Cortisol levels during embryogenesis were analysed by two-way ANOVA. A Tukey post hoc test was used to determine treatment and time effects. All other data were assessed by Student’s t-tests (unpaired). Data were transformed where necessary to meet the normality and equal variance assumptions of parametric data, and if these assumptions could not be met, a non-parametric test was carried out (Mann Whitney U test). The significance level (α) was set to 0.05, and SigmaPlot 13 (Systat Software, Inc.) was used for all statistical analyses.

## Additional Information

**How to cite this article:** Best, C. *et al*. Maternal cortisol stimulates neurogenesis and affects larval behaviour in zebrafish. *Sci. Rep.* **7**, 40905; doi: 10.1038/srep40905 (2017).

**Publisher's note:** Springer Nature remains neutral with regard to jurisdictional claims in published maps and institutional affiliations.

## Supplementary Material

Supplemental Information
