# Supplementary material for: Maternal cortisol stimulates neurogenesis and affects larval behaviour in zebrafish
Source: Sci Rep. 2017 Jan 18;7:40905. doi: 10.1038/srep40905 (PMC5241638; doi:10.1038/srep40905)

Supplementary Information

Maternal cortisol stimulates neurogenesis and affects larval behaviour in zebrafish

Carol Best[1], Deborah M. Kurrasch[2] and Mathilakath M. Vijayan[1,*]

[1]Department of Biological Sciences and
[2]Department of Medical Genetics
University of Calgary
2500 University Drive NW
Calgary, Alberta
Canada, T2N 1N4

***Corresponding Author**

Email: matt.vijayan@ucalgary.ca

Tel: +1 403 220 3094

Supplementary Information

SI Table 1.

List of abbreviations for brain regions.

**Neurogenesis (EdU) - Immunohistochemistry**			
ac	anterior commissure	poc	postoptic commissure
DT	dorsal thalamus	Pr	pretectum
EmT	eminentia thalami	PTd	dorsal part of posterior tuberculum
H	hypothalamus	PTv	ventral part of posterior tuberculum
MO	medulla oblongata	S	subpallium
OB	olfactory bulb	T	midbrain tegmentum
oc	optic chiasma	TeO	tectum opticum
P	pallium	TeVe	tectal ventricle
Po	preoptic region	VT	ventral thalamus (prethalamus)
***In Situ* Hybridization**			
H	hypothalamus		
HB	hindbrain		
MB	midbrain		
Ret	retina		

SI Table 2.

Primer sequences for riboprobe synthesis. Primer sequences and product sizes for oligonucleotide primers used in template generation by PCR for riboprobe synthesis for neuronal differentiation 4 (neurod4, NM_170762.1) and orthopedia b (otpb, NM_131100.1). T7 RNA polymerase binding sites are indicated in italics.

**Gene (amplicon)**	**Probe**	**Primers (5' to 3')**
neurod4 (923 bp)	sense	F: *TAATACGACTCACTATAGGG*CAGGTGCTACAATAACAGATCACA
		R: GTAGTGAGTCGGATGAGGCG
	antisense	F: CAGGTGCTACAATAACAGATCACA
		R: *TAATACGACTCACTATAGGG*GTAGTGAGTCGGATGAGGCG
otpb (537 bp)	sense	F: *TAATACGACTCACTATAGGG*TCAACTCCACTGGATGAAGGATG
		R: AGACGGGAACTGAGGCAAAC
	antisense	F: TCAACTCCACTGGATGAAGGATG
		R: *TAATACGACTCACTATAGGG*AGACGGGAACTGAGGCAAAC

SI Figure 1.

Neurogenesis in the rostral hypothalamus, posterior tuberculum, and dorsal thalamus in larvae with altered cortisol deposition. Larvae were pulsed with EdU at 24 hpf and then raised until 5 dpf. A representative image as well as an overlay of staining is shown for each region quantified (control), with a representative image from the cortisol-treated group (A). Regions quantified are (B) rostral hypothalamus, (C) posterior tuberculum, and (D) dorsal thalamus. Red is EdU staining, green is HuC staining and blue is DAPI staining. Values represent means ± SEM, and different letters indicate significant differences (Student's t-test, unpaired, n=4-5).

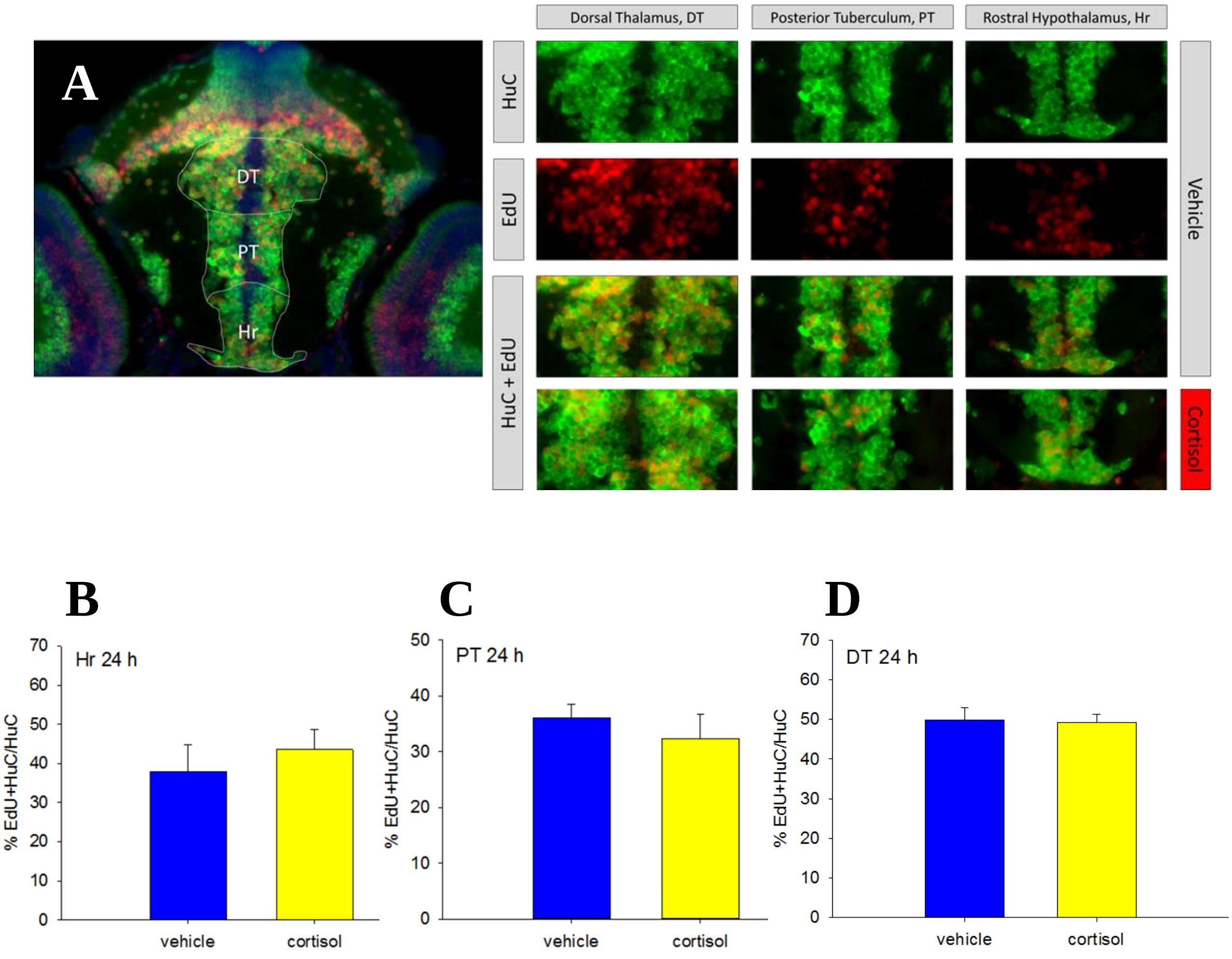

Supplement: Supplemental Information [file srep40905-s1.pdf]
